# 

**Published:** 1855-10

**Authors:** 


*Notice to Delinquents.*—It is very necessary that all accounts accruing under the former proprietorship of the Journal should be adjusted early during the present month. All our friends know the amount of their indebtedness to June 1st, 1855, and we urgently request those in arrears more than two years, to do us the justice to remit by mail, at our risk. We have very many accounts running back for a disreputable number of years.

